# Deposited ultra-thin titanium nitride nanorod array as a plasmonic near-perfect light absorber

**DOI:** 10.1038/s41598-020-79399-4

**Published:** 2020-12-17

**Authors:** Yi-Jun Jen, Kai-Bin Yang, Po-Chun Lin, Meng-Hsun Chung

**Affiliations:** Department of Electro-Optical Engineering, National Taipei University of Technology, Taipei, 106 Taiwan

**Keywords:** Nanowires, Metamaterials, Structural properties

## Abstract

The transmittance, reflectance, and extinctance that correspond to the localized plasmonic resonance within TiN nanorods were investigated. The obliquely deposited TiN nanorod array shows polarization-independent admittance matching to air. Unlike noble metal nanorods, the near-field localized longitudinal and transverse plasmonic resonance of TiN nanorod arrays present polarization-dependent light extinction in the far field. The longitudinal plasmonic mode presents stronger extinction than transverse plasmonic mode. In order to have high efficient light absorption, an ultra-thin two-layered TiN nanorod array was fabricated with orthogonal deposition planes for upper layer and bottom layer to absorb different polarized light energy. The measured spectrum shows broadband and wide-angle light extinction.

## Introduction

Perfect optical absorbers have been rapidly developed for more than a decade; they have many applications in photovoltaic^1,2^, sensing^3,4^, radiative cooling^5^, thermal light sources^6^. Ten years ago, a perfect light absorber was developed for the broadband and omnidirectional absorption of light; it relies on broadband and wide-angle antireflection to couple most incident light into a nanostructure to dissipate light energy^7–10^. To achieve perfect antireflection, Dobrowolski^11^ proposed the use of a grade-index profile to eliminate the reflection that occurs at an interface that has an abrupt index change. The grade-index profile requires the structure to be made of dielectric materials with ultra-small extinction coefficients. The biomimic silicon nanotip array, glancing angle-deposited five-layered structure and carbon nanotube array provide a grade-index profile to approach perfect light absorption^12^. The large thickness requirement can be eliminated by designing and fabricating a metamaterial as a light absorber. Aydin et al.^13^ used a multi-resonant metamaterial that was based on silver to achieve a measured absorption of 0.71 for visible wavelengths. Emerging metamaterials are versatile in light harvesting and have high efficiency. Yi-Jun et al.^14^ developed a seven-layered symmetrical film stack that comprised Ta_2_O_5_, Ge, Cr and Al as an equivalent layer with tailored admittance of close to unity and a refractive index with a large extinction coefficient, achieving an absorptance of 92% over a wide range of wavelengths of light, 400–2000 nm.

As well as having a compact structure, light absorbers that are used in harvesting energy must refract stably and be chemically stable. One of their important applications in solar thermophotovoltaics (STPV) has recently been intensively studied^15,16^. A thermophotovoltaic system includes a broadband absorber that collects solar energy efficiently to deliver sufficient heat to a selective emitter. The high operating temperature of thermovoltaic devices prevents the use of noble metals in plasmonic metamaterial absorbers. Transition metal nitrides such as titanium nitride (TiN) and zirconium nitride (ZrN) were recently identified as alternative plasmonic materials^17–19^. With high melting points and chemical stability at temperatures above 2900 °C, TiNs have a mechanical refractory property and the same optical property as noble metals. A three-layered TiN square ring array/SiO_2_ thin film/TiN film as a metamaterial absorber^20^ with a thickness of 240 nm has been found to have an absorptance maximum of around 100% at a wavelength of 650 nm, decaying to 87% at 800 nm. However, the relation between near-field localized plasmonic resonance and far-field transmission and reflection is yet to be investigated. On the other hand, perfect absorption by a TiN is expected to extend to the infrared range.

A sputtering system can be used directly to fabricate TiN films^21,22^. The permittivity of TiN film can be tuned by controlling the deposition parameters, including the substrate bias voltage and the nitrogen and argon flow rates. However, the extraordinary properties of TiN depend on its nanostructure, which is responsible for its subwavelength plasmonic resonance. Glancing angle deposition in a sputtering system can easily be used to fabricate TiN nanorod arrays. Sputtered atoms are aligned and the flux is controlled by setting a plate parallel to the substrate^23^. The grains that are initially deposited on the substrate form a shadow with respect to the incoming vapor flux so the flux atoms that are deposited on the grains obliquely form nanorods. The absorption of a glancing angle-deposited TiN nanorod array (NRA) has been demonstrated to vary with the deposition parameters^24^.

In this work, a two-layered TiN nanorod array is developed as a perfect light absorber. This obliquely deposited TiN nanorod array exhibits polarization-independent admittance matching with air. Based on analysis of the polarization-dependent absorption, which is related to the longitudinal plasmon resonance and transverse plasmon resonance^25,26^, two-layered TiN nanorod arrays with different directions of growth are stacked to perform broadband and wide-angle light absorption.

## Results

**Figure 1:**
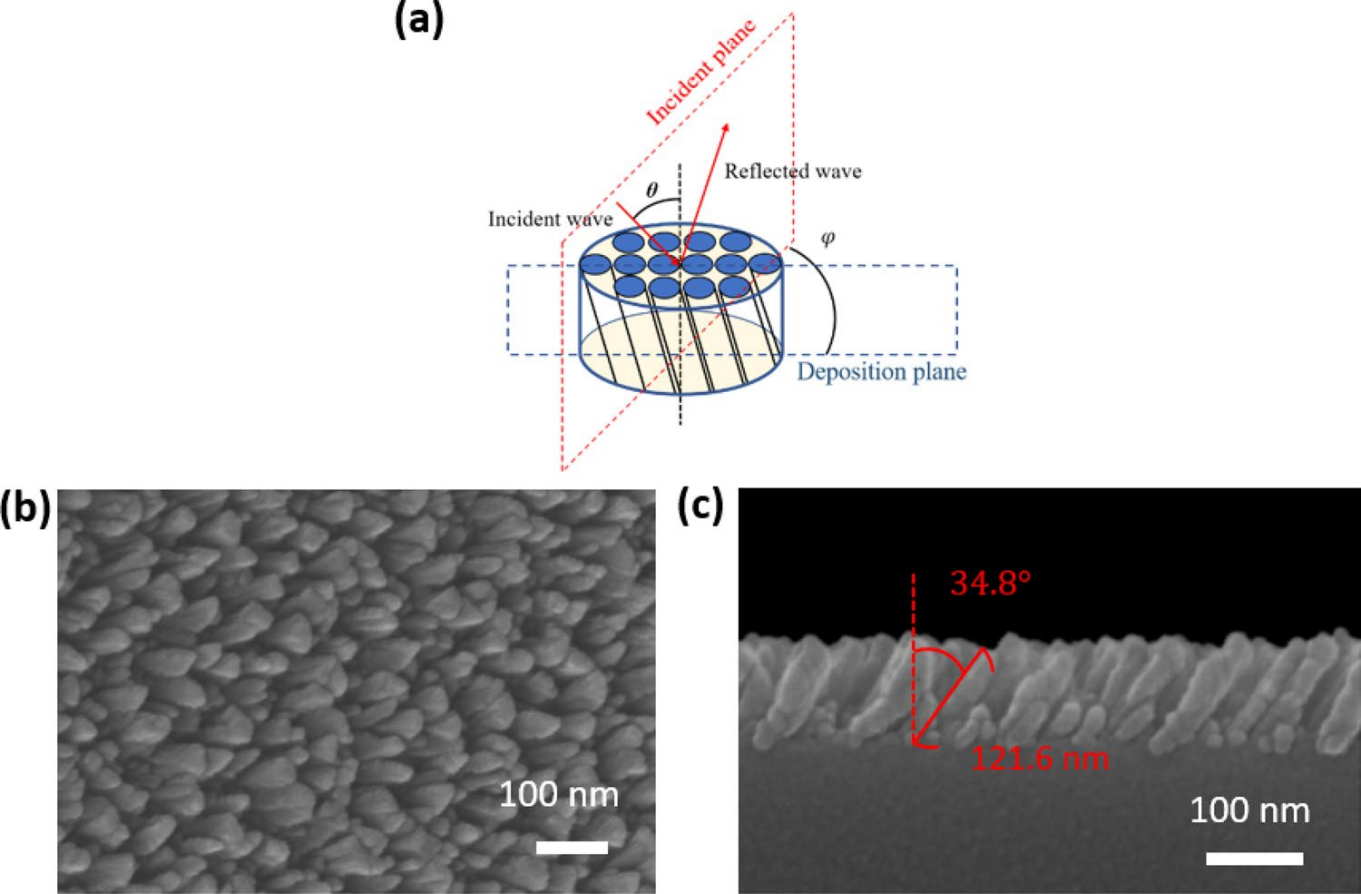
(**a**) Orientations of nanorods with respect to the plane of incidence; (**b**) top-view and (**c**) cross-sectional SEM images of TiN NRA.

**Figure 2:**
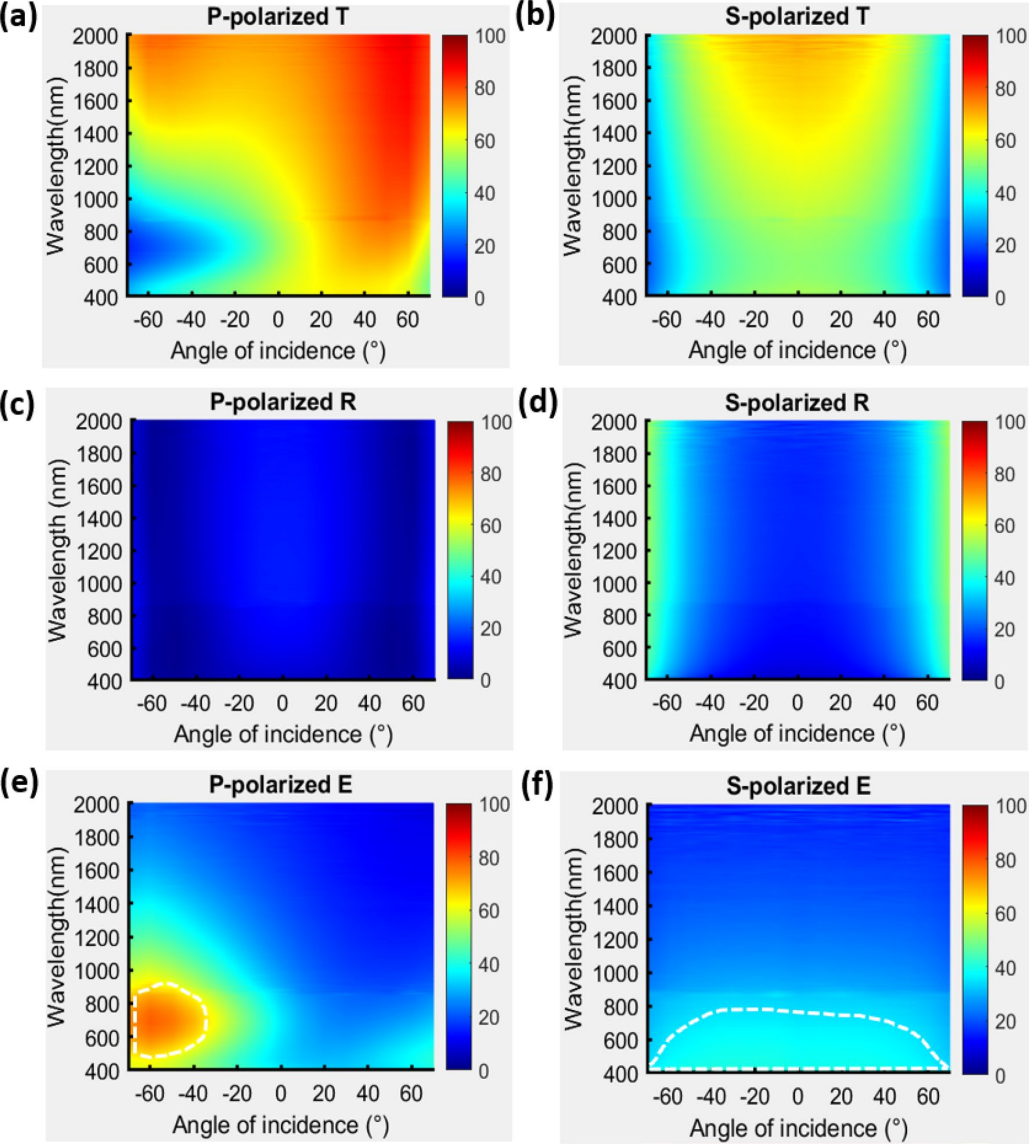
P-polarized and s-polarized transmittance (**a**,**b**), reflectance (**c**,**d**) and extinctance (**e**,**f**) spectra measured at $\varphi = 0^\circ$ that incident plane is coincident with deposition plane.

With the deposition parameters that are mentioned in the section of methods, a uniform TiN film was deposited and its permittivity spectrum was measured; the spectrum is shown in the supplementary information. The single TiN NRA was deposited at an angle of $84^\circ$ between the vapor flux and substrate surface. As shown in Fig. 1, the average tilt angle between the rods and the normal to the substrate surface was $\beta = 34.8^\circ$. The deposition plane is the plane that contains the growth direction of the nanorods and the surface normal. The angle of incidence is ${\uptheta }$ and the angle between the deposition plane and incident plane is $\varphi$. The average width and length of TiN NRAs was estimated from the cross-sectional SEM images by using an image processing program (Image J, 1.48). The widths of rods were measured at the middle of each rod. The width and length of the rods were 26.4 nm and 121.6 nm, respectively. The porosity of the TiN NRA was approximately 25.2%. The polarization-dependent reflectance (R) and transmittance (T) spectra were measured at angles of incidence that were integer multiples of $10^\circ$, with the plane of incidence coincident with the deposition plane, as shown in Fig. 2. The TiN nanorod array exhibits admittance matching with the air. Although the nanorods are tilted with respect to the surface normal, the p-polarized transmittance spectrum is asymmetrical about the surface normal but the s-polarized transmittance spectrum is almost symmetrical about the surface normal, as shown in Fig. 2a,b. The p-polarized reflectance is less than 10% over all wavelengths from $\lambda = 400 \;{\text{nm}}$ to $\lambda = 2000\;{\text{nm}}$ and angles of incidence from $\left| \theta \right| = 30^\circ$ to $\left| \theta \right| = 60^\circ$, as shown in Fig. 2c. At any wavelength, the reflectance has a maximum at $\theta = 0^\circ$ and decays to a minimum at $\theta = \pm \;60^\circ$. At $\theta = \pm \;60^\circ$, the reflectance is less than 4.67% over the whole range of wavelengths. The average p-polarized reflectance over the wavelengths and incident angles is 8.08%. The s-polarized reflectance is less than 17.66% at angles of incidence from $\theta = - \;40^\circ$ to $\theta = 40^\circ$, revealing good admittance matching over the wavelength range from $\lambda = 400\;{\text{nm}}$ to $\lambda = 500 \;{\text{nm}}$, as shown in Fig. 2d. The reflectance increases smoothly from $\lambda = 400 \;{\text{nm}}$ to $\lambda = 2000 \;{\text{nm}}$ at angles between $\theta = 40^\circ$ and $\theta = - \;40^\circ$. Unlike the p-polarized reflectance, the s-polarized reflectance has a minimum at angle of incidence $\theta = 0^\circ$, increasing with $\theta$, as is conventional for s-polarized reflection at an interface. The average s-polarized reflectance over the wavelengths and incident angles is 26.16%.

**Figure 3:**
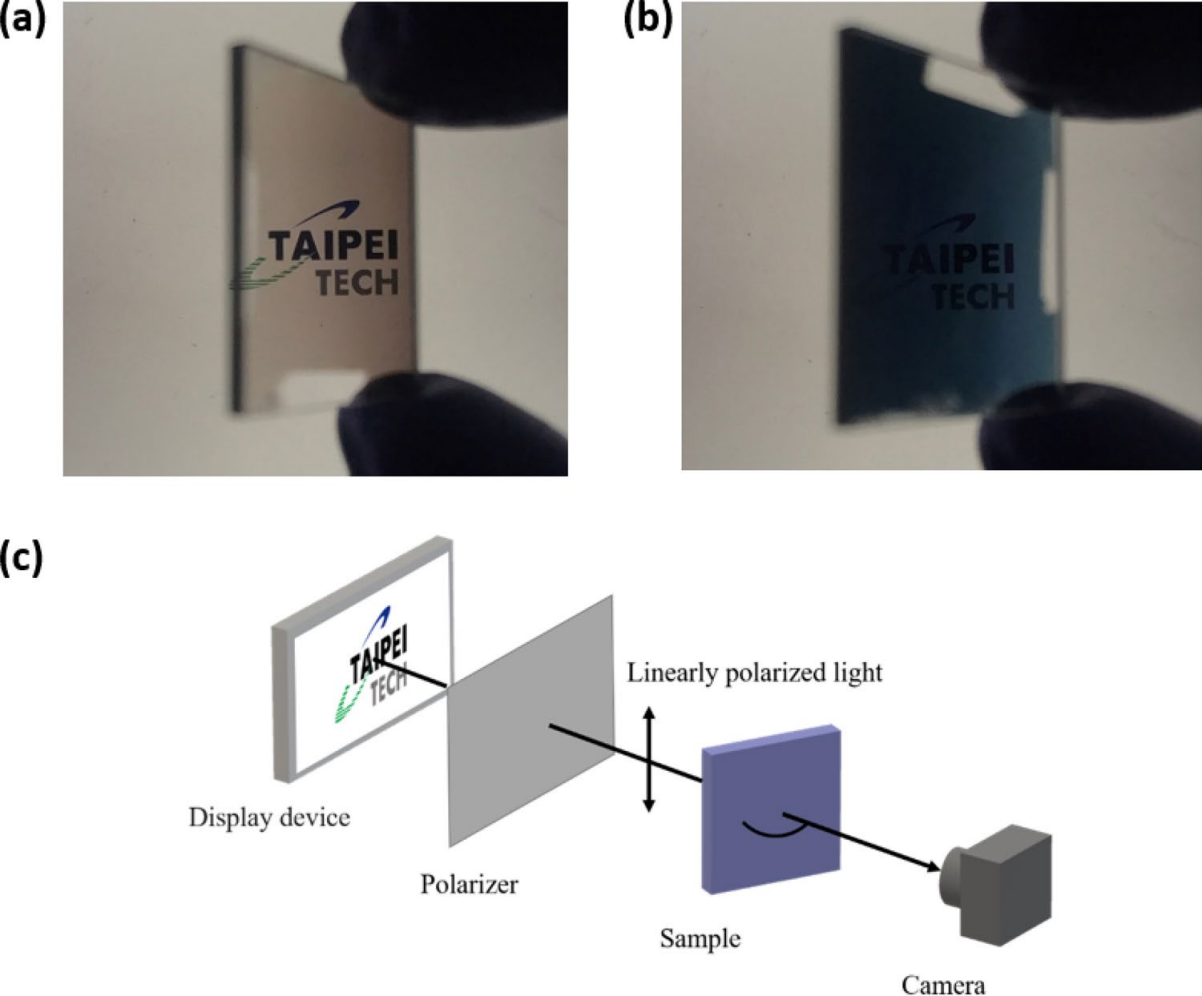
P-polarized transmitted images captured at (**a**)$(\varphi ,\;\theta ) = (0^\circ ,\;60^\circ )$ and (**b**)$(\varphi ,\;\theta ) = (0^\circ ,\; - \;60^\circ )$; (c) Setup for image capture.

Figure 2e,f show the extinctance E, derived from E = 1 − R − T^27,28^. The p-polarized extinctance has a high value of E = 78.1% at $\theta = - \;60^\circ$ and $\lambda = 685 \;{\text{nm}}$; in this direction, the electric field oscillates along the rods, inducing longitudinal plasmon resonance. The p-polarized extinctance of over 65% is continuously distributed in the area inside the white dash lines in Fig. 2e. When an s-polarized incident wave is incident on a tilted nanorod array, the electric field usually oscillates perpendicular to the rods at all angles of incidence, and transverse plasmon resonance is induced over a wide range of angles at a wavelength of close to 400 nm. Therefore, s-polarized extinctance is strong at 400 nm over a wide range of incident angles. An s-polarized extinctance of over 35% is continuously distributed in the area inside white dash lines in Fig. 2f. Figure 3 shows the p-polarized transmission images that were captured at $\theta = 60^\circ$ and $\theta = - \;60^\circ$. The image at $\theta = 60^\circ$ was blocked and the image at $\theta = - \;60^\circ$ was clearly captured. Interestingly, the localized plasmonic resonances within the TiN nanorods present polarization-dependent absorption, unlike the nobel metal nanorod arrays. The longitudinal and transverse plasmon resonances of an obliquely silver nanorod array reveal not only polarization-dependent absorption but also obvious polarization-dependent reflection^29^.

**Figure 4:**
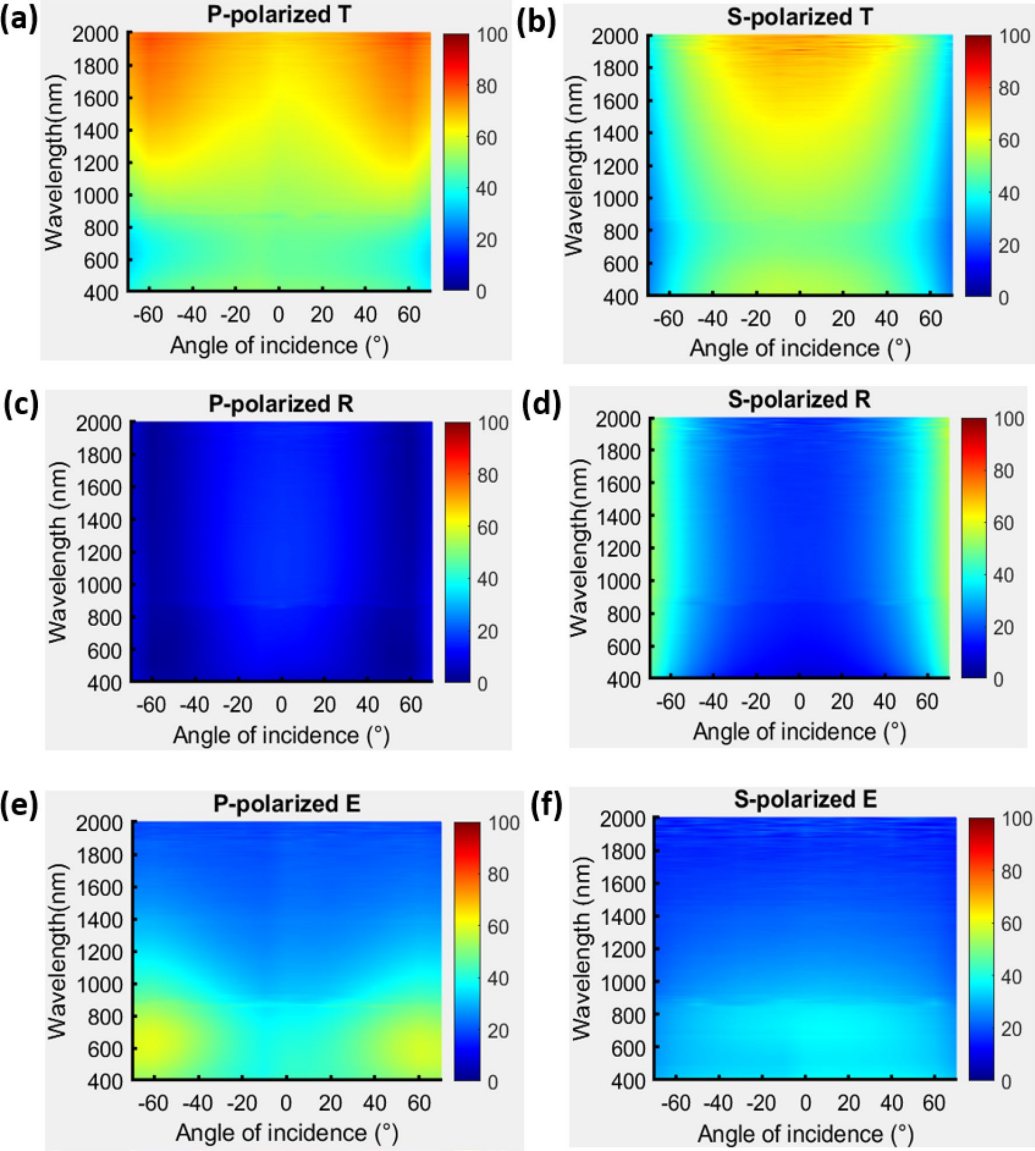
P-polarized and s-polarized transmittance (**a**,**b**), reflectance (**c**,**d**) and extinctance (**e**,**f**) spectra measured at $\varphi = 90^\circ$ that incident plane is perpendicular to deposition plane.

The polarization-dependent extinction is further investigated by measuring the reflectance and transmittance spectra with the plane of incidence perpendicular to the deposition plane ($\varphi = 90^\circ$), as shown in Fig. 4. The p-polarized and s-polarized reflectance spectra are similar to the spectra that were measured at $\varphi = 0^\circ$, exhibiting low reflection over a broadband and a wide range of angles. The s-polarized extinction is symmetrical about the surface normal and yields a spectrum similar to that in the case of $\varphi = 0^\circ$. Since the electric field of p-polarized light has a large component perpendicular to the rods at all angles, the p-polarized extinctance is nearly symmetrical about the surface normal and has two maxima at $\theta = 60^\circ$ and $\theta = - 60^\circ$ at 630 nm, corresponding to the longitudinal plasmonic mode.

The extinctance over visible wavelengths can be increased by increasing the thickness of TiN NRA. A 281 nm-thick TiN NRA that is fabricated with the same deposition parameters has higher extinction, as shown in Fig. S2 and Fig. S3. A thicker TiN NRA exhibits stronger light extinction over the visible wavelengths, with a highest extinctance of 97.6% at $(\lambda ,\;\theta ) = (679\;{\text{nm}},\; - \;50^\circ )$) when the incident plane is oriented at $\varphi = 0^\circ$. The extinctance is reduced to be less than 30% at 2000 nm, favoring the prevention of radiation loss in some STPV systems^30^.

The reflectance, transmittance and extinctance spectra of a uniform TiN film with a thickness of 100 nm as the aforementioned TiN NRA are plotted in Fig. S4 with the permittivity function shown in Fig. S1. The sample is opaque and strong light absorption only occurs at wavelengths near 400 nm. The reflectance exceeds 60% for wavelengths over 813 nm at normal incidence. As most optical coatings, the s-polarized reflectance increases with angle of incidence and larger than the p-polarized reflectance.

**Figure 5:**
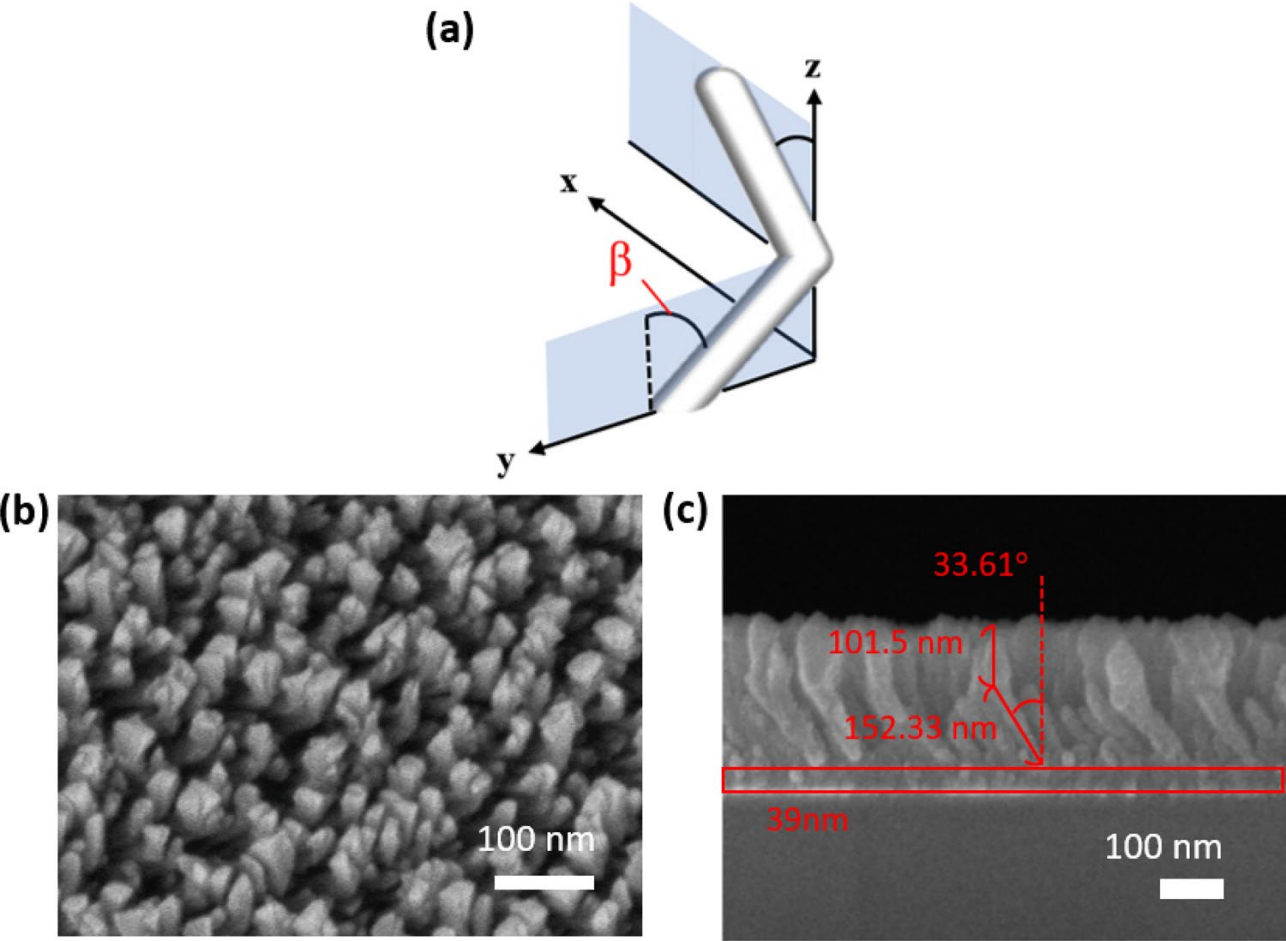
(**a**) Orientations of nanorods with respect to system coordinates. (**b**) Top-view and (**c**) cross-sectional SEM images of two-layered TiN NRA.

**Figure 6:**
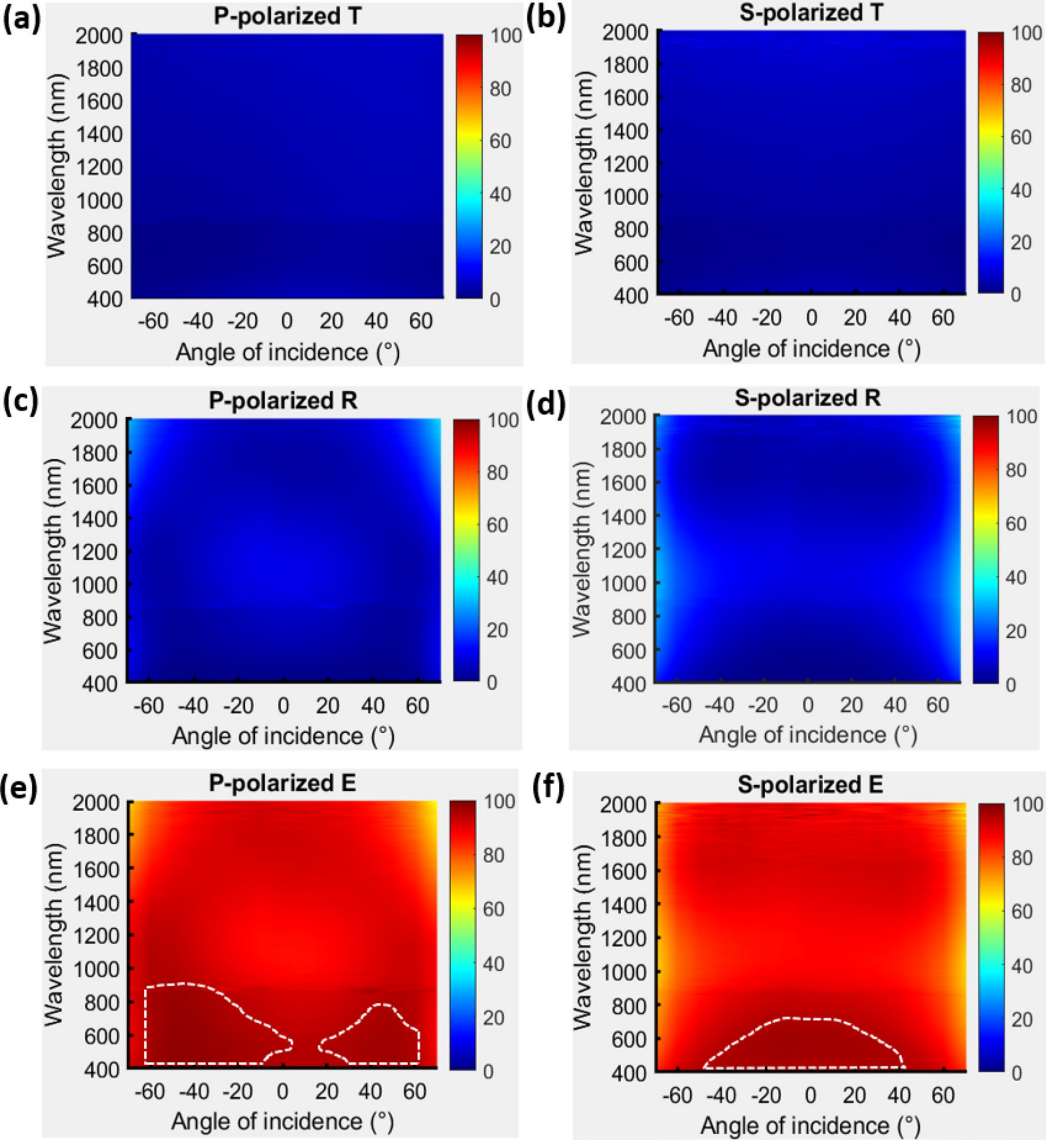
P-polarized and s-polarized transmittance (**a**,**b**), reflectance (**c**,**d**), and extinctance (**e**,**f**) spectra.

Based on the aforementioned analysis, a two-layered TiN nanorod array as a broadband and wide-angle light absorber is developed here. As shown in Fig. 5a, the upper NRA was deposited by rotating the substrate by $90^\circ$ following the deposition of the bottom nanorod array, so the deposition plane of the upper NRA was perpendicular to that of the bottom NRA. The two TiN NRAs were stacked on a 39 nm-thick uniform TiN thin film that was deposited on a BK7 substrate. The top-view and cross-sectional SEM images are shown in Fig. 5b,c, respectively. The uniform TiN film was used to reduce the transmission and reflect light back to the nanorods to dissipate its energy again. The average length, rod width and tilt angle of the upper NRA were 127.8 nm, 49.7 nm and $37.4^\circ$, respectively. The average length, rod width and tilt angle ${\upbeta }$ of the bottom NRA were 152.3 nm, 33.1 nm and $33.6^\circ$, respectively. The total thickness of the TiN structure was 232.3 nm. Figure 5a also schematically depicts the orientations of the nanorods with respect to the system coordinates. The s-polarized and p-polarized transmittance, reflectance and extinctance spectra were measured with the incident plane coincident with the x–y plane. The p-polarized extinctance was larger than 90% at angles of incidence from $0^\circ$ to $- \;60^\circ$ and wavelengths from $\lambda = 400 \;{\text{nm}}$ to $\lambda = 861 \;{\text{nm}}$. For wavelengths of greater than 1400 nm, a high extinctance of over 78.9% obtained at angles of incidence between $- \;50^\circ$ and $50^\circ$. For wavelengths from 900 to 1300 nm, the reflectance versus angle of incidence had a maximum at normal incidence, decaying to a minimum at angles around $- \;50^\circ$—similar to conventional reflection at the Brewster angle. High extinctance from 400 to 851 nm has values of over 90.8% between angles $- \;60^\circ$ and $60^\circ$. At wavelengths above 1400 nm, the extinctance has a maximum around 93.8% and decays as the angle of incidence increases. Of the two strong extinctance areas on the $\theta$–${\uplambda }$ plane, one is centered at $(\lambda ,\;\theta ) = (498\;{\text{ nm}},\;50^\circ )$ with an extinctance peak of 98.4% and the other is centered at $(\lambda ,\;\theta ) = (506\; {\text{nm}},\;50^\circ )$ with an extinctance peak of 96.9%. Both areas in which the p-polarized extinctance exceeds 95% are indicated by a white dashed line in Fig. 6.

The s-polarized extinctance has a strong band from 400 to 800 nm. The angular range that is associated with the strong extinctance shrinks gradually as the wavelength increases. At 400 nm, the extinctance exceeds 91.9% from $- \;60^\circ$ to $60^\circ$. At 800 nm, the extinctance exceeds 86.4% from $- \;50^\circ$ to $50^\circ$. The extinctance is uniformly distributed between 400 and 800 nm. At wavelengths of over 1400 nm, the extinctance rises to maximum values of 93.2% at $(\lambda ,\; \theta ) = (1740 \;{\text{nm}},\; - \;30^\circ )$ and 92.6% at $(\lambda ,\;\theta ) = (1617 \;{\text{nm}},\;40^\circ )$. The s-polarized transmittance is 8–13.9% and the p-polarized transmittance is 3.2–7.3% at wavelengths above 1400 nm. A thicker uniform TiN layer underneath the nanostructured TiN films provides greater extinctance by reducing the transmittance. The extinctance versus $\theta$ and $\lambda$ has a peak at $(\lambda ,\;\theta ) = (566 \;{\text{nm}},\; - \;10^\circ )$ with a maximum value of 97.0%. The s-polarized extinctance exceeds 95% in the area that is marked with a white dashed line in Fig. 6f.

## Discussion

**Figure 7:**
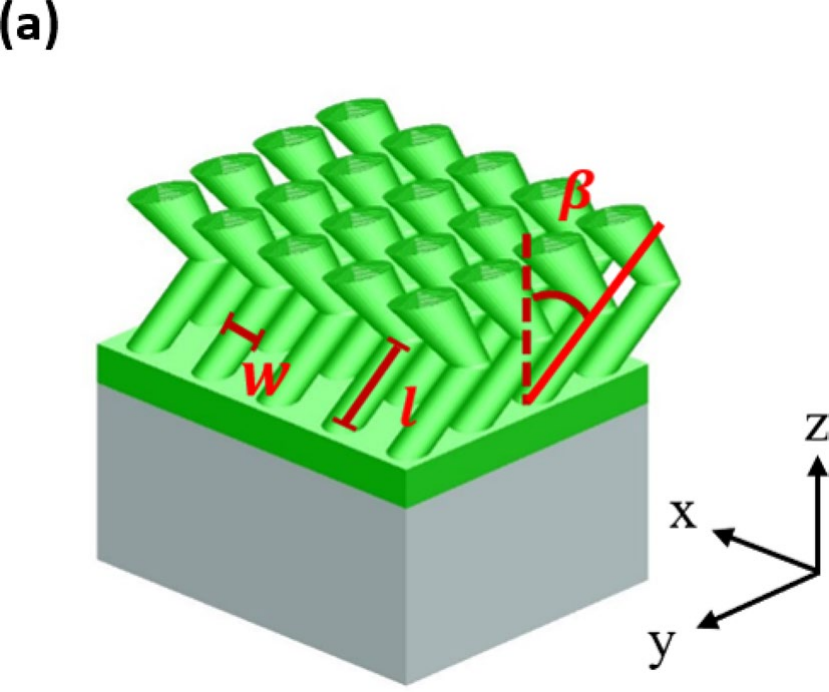
A truncated view of the two-layered TiN NRA on a 39 nm-thick uniform TiN film for simulation. The substrate is a BK7 glass.

The finite-difference time-domain (FDTD) (Lumerical FDTD Solutions 8.11.337) is adopted to simulate the near-field electric field within the two-layered structure. As shown in Fig. 7, identical TiN nanorods with geometric parameters of ($\beta$, w, l) = (30°, 32.5 nm, 140 nm) are regularly distributed as the bottom NRA and the center-to-center distance between adjacent rods is 70 nm. The upper nanorods are connected to the bottom rods with deposition plane on x–z plane that is perpendicular to that of the bottom rods. A 39 nm-thick TiN film is arranged between the two-layered NRA and a BK7 glass substrate. Since the fan-out phenomenon observed from the SEM is obvious for the upper layer, the rod width is set to increase from 16.2 to 29.3 nm linearly. The length and tilt angle of upper nanorods are 117 nm and 30°, respectively. The permittivity of the TiN is adopted from functions of the real part and imaginary part of the permittivity shown in Fig. S1.

**Figure 8:**
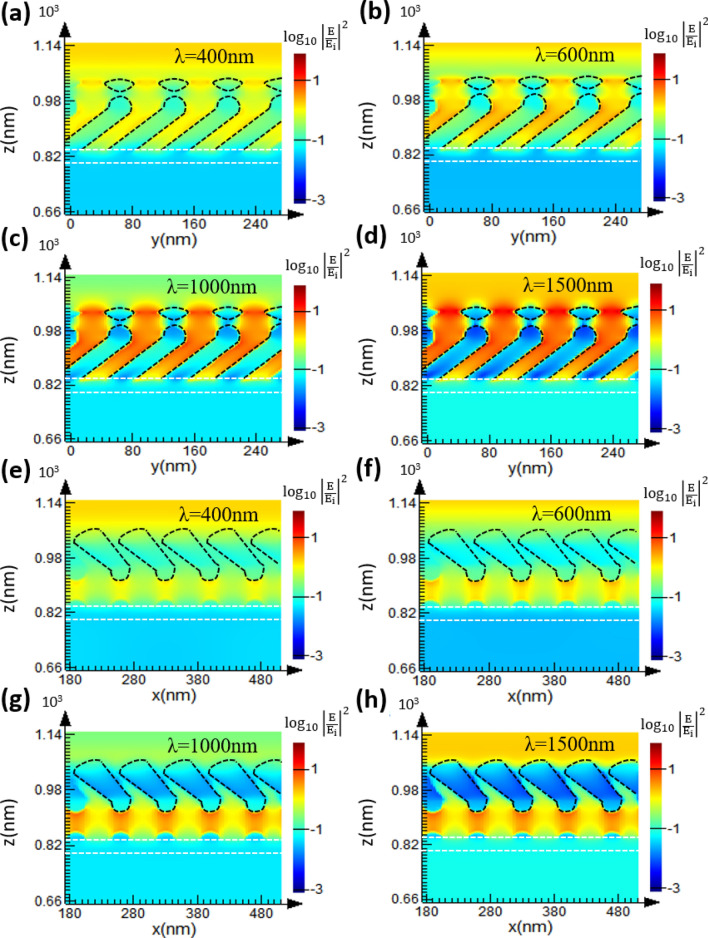
(**a**–**d**) Electric field intensity distributions for the y–z plane across the center of bottom rods; (**e**–**h**) Electric field intensity distributions for the x–z plane across the center of upper rods.

**Figure 9:**
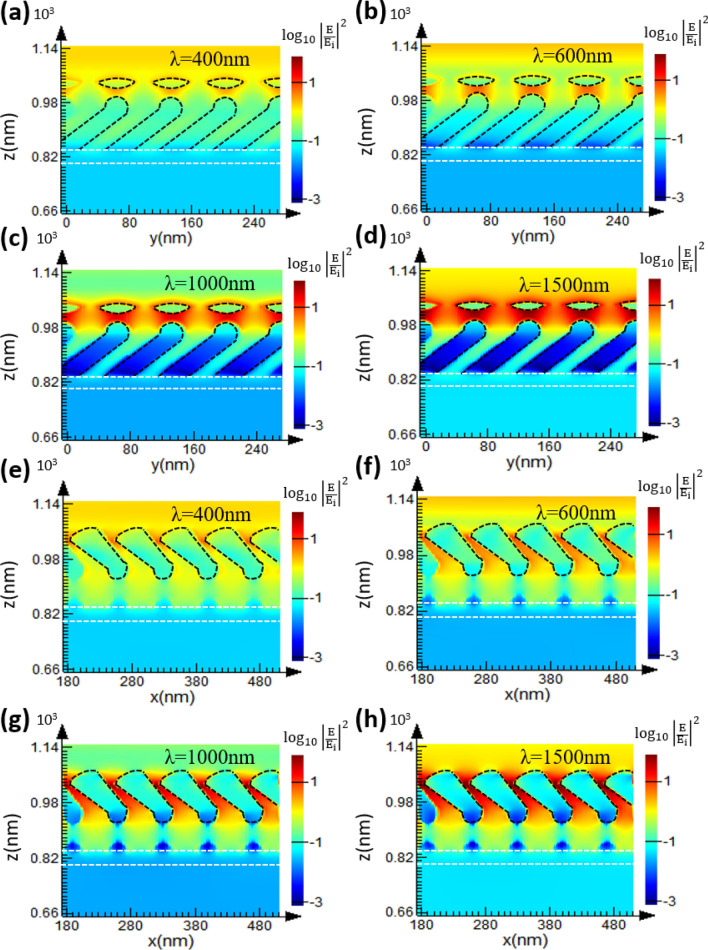
(**a**–**d**) Electric field intensity distributions for the y–z plane across the center of bottom rods; (**e**–**h**) electric field intensity distributions for the x–z plane across the center of upper rods.

The electric field intensity distributions are simulated at wavelengths of 400 nm, 600 nm, 1000 nm and 1500 nm. The electric field intensity defined as $\left| {{\text{E}}/{\text{E}}_{{\text{i}}} } \right|^{2}$ where E_i_ and E are the amplitudes of incident electric filed and localized electric field, respectively. For p-polarization (the electric field is oscillating along the x-axis), the cross-sectional x–z and y–z planes shows that the bottom rods have stronger field enhancement than the upper rods, as shown in Fig. 8. The strong electric filed distributed inside each rod indicates the resonance of transverse plasmonic mode^26^. For s-polarization (the electric field is oscillating along the x-axis), the cross-sectional x–z and y–z planes shows that the upper rods have stronger field enhancement than the bottom rods, as shown in Fig. 9. The strong electric filed distributed between rods indicates the resonance of longitudinal plasmonic mode^26^. Since the permittivity of the TiN film is negative and its magnitude increases with wavelength, the optical property of the TiN film is similar to noble metals at infrared wavelengths. Therefore, the enhanced field intensities at wavelengths of 1000 nm and 1500 nm are stronger than those at 400 nm and 600 nm. The metal-like optical property of TiN NRA would enhance longitudinal plasmonic resonance and lead to increase in reflection. Therefore, the extinction at visible wavelengths is larger than that at infrared wavelengths.

In summary, a broadband absorber was designed, fabricated and optically characterized. The admittance matching of an obliquely deposited TiN nanorod array reveals that the localized plasmon resonance exhibits absorption only in the far field. By varying the orientation of a TiN nanorod array with respect to the incident polarized light, the absorption spectrum can be varied to fulfill certain demand. Since TiN is a refractory material, the TiN nanorod absorber can be applied in an STPV system to absorb strongly visible and NIR wavelengths. A two-layered TiN nanorod array in this work exhibits broadband and wide angle light absorption. As the TiN nanorod array or TiN film underneath the nanorods becomes thicker, the absorption will increase furthermore. This work proposes an easy method for the bottom-up nanostructure fabrication on a large area. Since the optical and mechanical properties of TiN films fabricated in a sputtering system can be varied by changing the reaction gas flow rate, substrate temperature and substrate bias voltage. TiN nanorods can be tuned in design and fabrication for use as an absorber with desired optical characteristics.

## Methods

The TiN film and TiN NRAs were deposited on BK7 glass substrates using a homemade DC reactive magnetron sputtering system with a 99.995% titanium target (Kurt Lesker, Jefferson Hills, PA, USA) in an argon–nitrogen environment. The base pressure in the chamber before deposition was 5 × 10^−6^ Torr. The films were deposited at a pressure of 2.8 × 10^−3^ Torr. The sputtering power was constant for all depositions at 200 W (DC). The deposition rate was around 1.0 Å/s and controlled with a quartz monitor. To deposit TiN, a pure Ti target was used with Ar as the sputtering gas and N_2_ as the reactive gas. To obtain stoichiometric TiN, the argon flow rate and nitrogen flow rate were maintained at 15 sccm and 1.2 sccm, respectively. The deposition angle of the stage is defined as the angle between the normal to the substrate and the direction of vapor flux from the sputtering source. The uniform TiN thin film was deposited firstly at a deposition angle of $0^\circ$. Then the bottom TiN NRA was deposited at a deposition angle of 84°. In order to control the sputtering flux rate, a stainless steel plate was placed parallel to the substrate at a distance 10 mm from it. After the deposition of bottom layer, the substrate was rotated by 90° to deposit the upper layer. The upper TiN NRA was grown at a deposition angle of 88° and the distance between the substrate and the stainless steel plate was 8 mm.

The permittivity spectrum was obtained using commercial spectroscopic ellipsometer (J. A. Woollam Co., VASE). The polarization-dependent reflectance (R) and transmittance (T) spectra were obtained using a UV–visible/NIR spectrophotometer (Hitachi High-Tech Corporation, UH4150).

## Supplementary Information

Supplementary Information.
