# Supplementary material for: Deposited ultra-thin titanium nitride nanorod array as a plasmonic near-perfect light absorber
Source: Sci Rep. 2020 Dec 17;10:22269. doi: 10.1038/s41598-020-79399-4 (PMC7747740; doi:10.1038/s41598-020-79399-4)
Supplement: Supplementary file 1 — Supplementary Information. [file 41598_2020_79399_MOESM1_ESM.docx]

**Supplementary information for: Deposited ultra-thin titanium nitride nanorod array as a plasmonic near-perfect light absorber**

**Yi-Jun Jen^1,^*, Kai-Bin Yang^1^, Po-Chun Lin^1^, and Meng-Hsun Chung^1^**

^1^Department of Electro-Optical Engineering, National Taipei University of Technology, Taipei 106, Taiwan

*****Correspondence: [jyjun@ntut.edu.tw](mailto:jyjun@ntut.edu.tw)


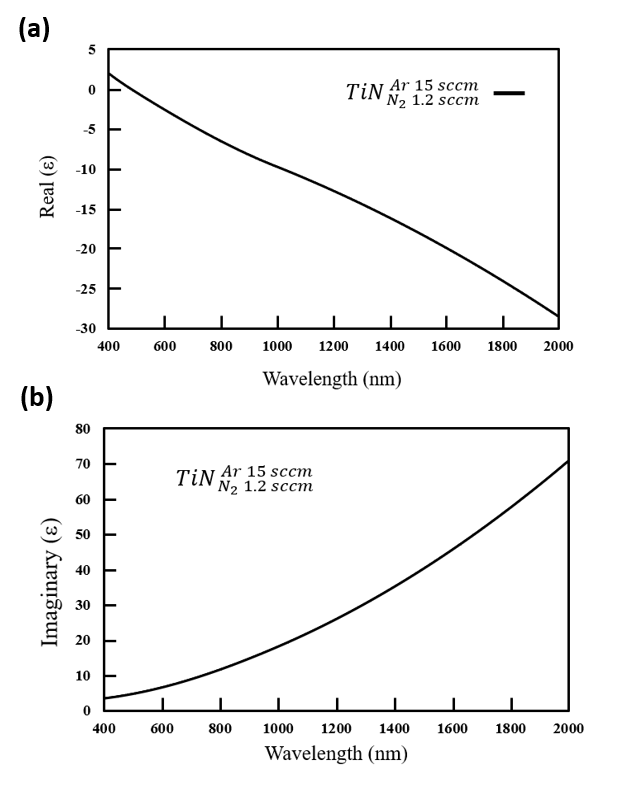


**Fig. S1**. (a) Real part and (b) imaginary part of permittivity ε for an uniform TiN film.


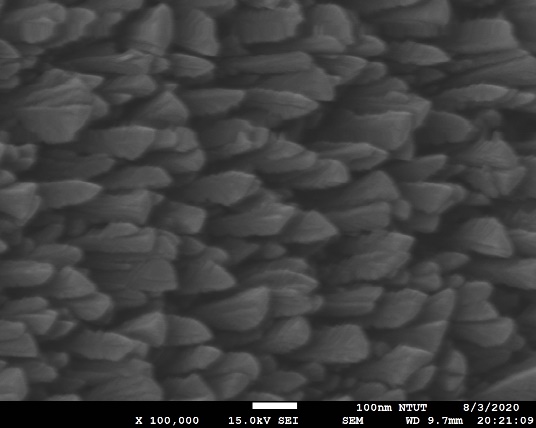


**(a)**

**(b)**


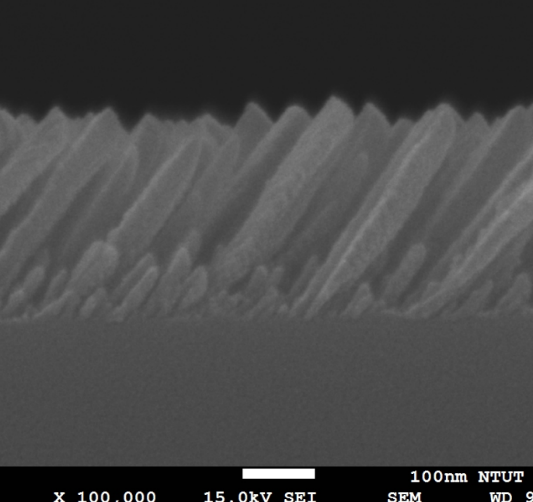


100nm

**Fig. S2.** (a) Top-view and (b) cross-sectional SEM images of TiN NRA.


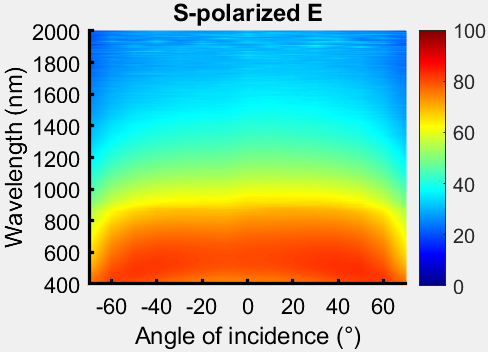


**(a)**

**(b)**

**(c)**

**(d)**

**(e)**

**(f)**


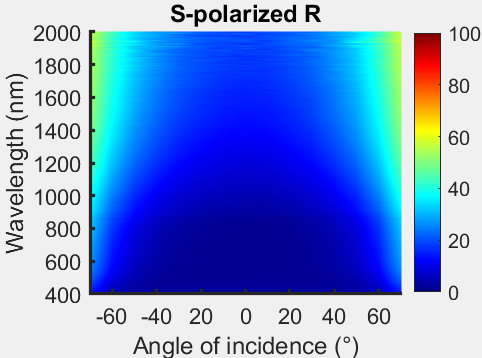

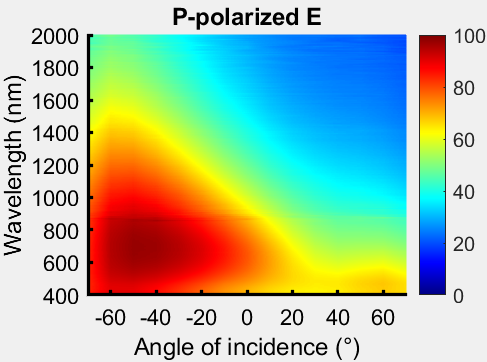

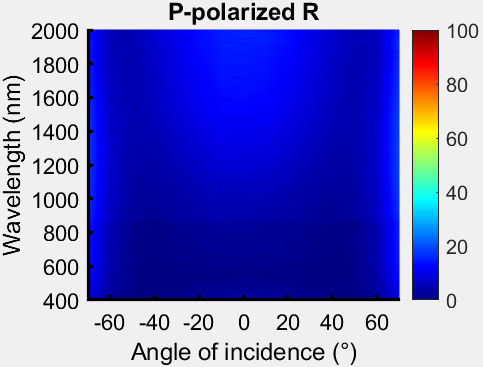

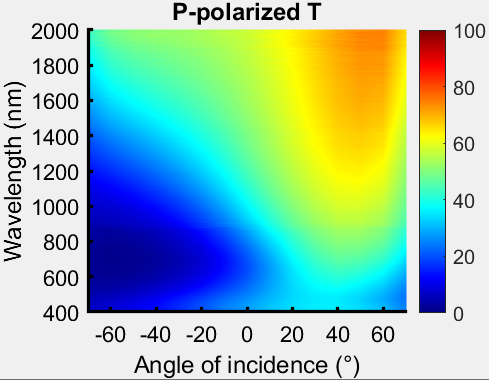

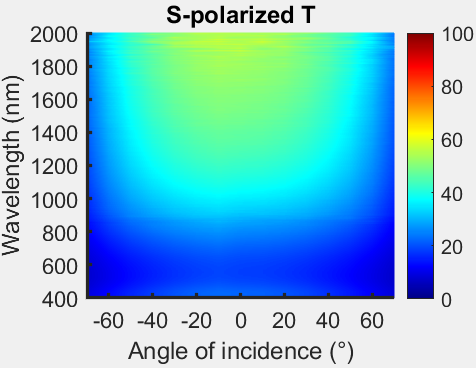


**Fig. S3.** P-polarized and s-polarized transmittance (a,b), reflectance (c,d) and extinctance (e,f) spectra at $\varphi=0^{\circ}$.


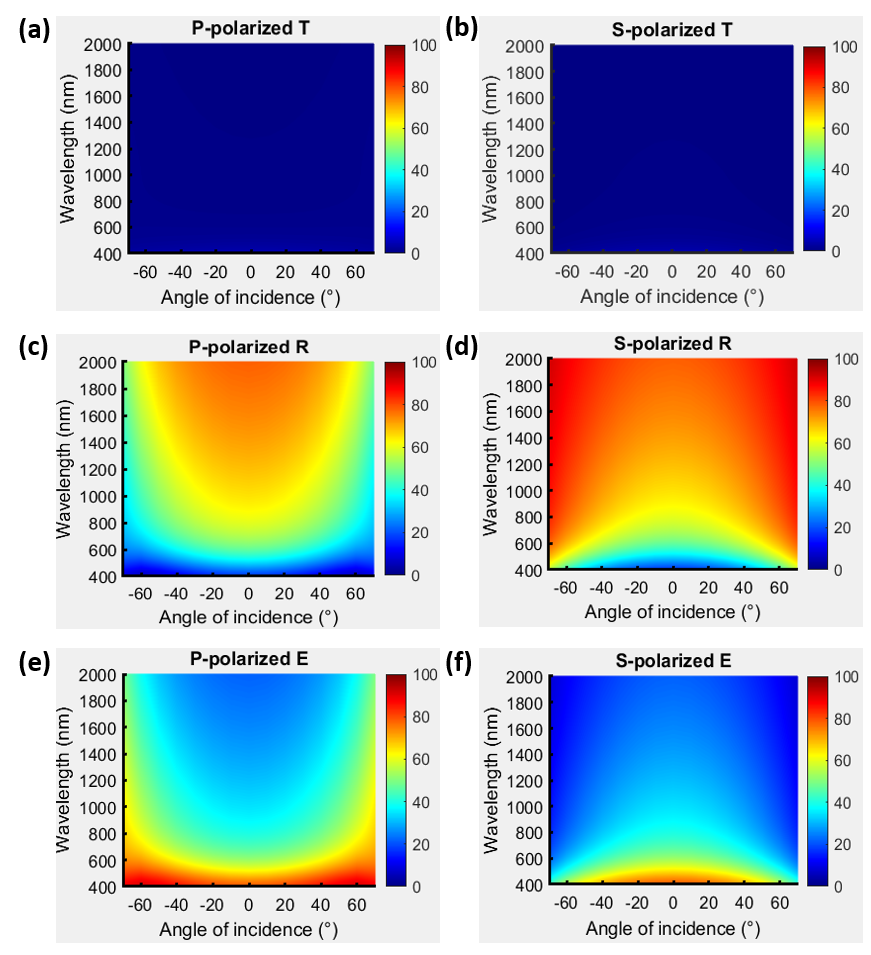


**Fig. S4.** P-polarized and s-polarized transmittance (a,b), reflectance (c,d) and extinctance (e,f) spectra of a uniform TiN film with a thickness of 100 nm.
